# Characterization of the complete chloroplast genome of *Fraxinus mandshurica* (Oleaceae)

**DOI:** 10.1080/23802359.2018.1532833

**Published:** 2018-10-26

**Authors:** Yuemei Zhao, Tao Zhou, Xiao Zhang, Xiaodan Chen, Guoqing Bai

**Affiliations:** a College of Biopharmaceutical and Food Engineering, Shangluo University, Shangluo, China;; b School of Pharmacy, Xi’an Jiaotong University, Xi’an, China;; c Key Laboratory of Resource Biology and Biotechnology in Western China (Ministry of Education), College of Life Science, Northwest University, Xi’an, Shaanxi Province, China;; d Shaanxi Engineering Research Centre for Conservation and Utilization of Botanical Resources, Xi’an Botanical Garden of Shaanxi Province, Xi’an, China

**Keywords:** *Fraxinus mandshurica*, chloroplast, Illumina sequencing, phylogenetic analysis

## Abstract

The whole chloroplast (cp) genome sequence of *Fraxinus mandshurica* has been characterized from Illumina pair-end sequencing. The complete cp genome was 155,530 bp in length, containing a large single copy region (LSC) of 86,415 bp and a small single copy region (SSC) of 19,279 bp, which were separated by a pair of inverted repeat (IR) regions of 25,653 bp. The genome contained 133 genes, including 88 protein-coding genes, 37 tRNA genes and eight ribosomal RNA genes (four rRNA species). Most genes occur as a single copy, while 19 gene species are duplicated. phylogenetic analysis revealed that *F. mandshurica* is closely related to the species of *F. chiisanensis* and *F. excelsior.*

*Fraxinus mandshurica* is a kind of dioecious, wind-pollinated and cold-tolerant tree species (Wu 1980; Kong 2004; Kong et al. 2008) belonging to the family Oleaceae. It is mainly distributed across Northeast China, in addition, there are some small populations in North-west China, the Russian Far East, Japan and North Korea (Wu 1980). Due to overexploitation and widespread deforestation during the past 50 years in China, the habitats of *F. mandshurica* have been severely decreased and fragmented, it has been listed as an endangered species (Fu 1992) and a national priority protected plant in China (Chinese Ministry of Forestry 1999). Therefore, it is urgently necessary to conserve the existing individuals of *F. mandshurica*. In this case, genomics resources are definitely fundamental to formulate a reasonable conservation strategy of an endangered species (O’Brien 1994). In this study, we assembled and characterized the complete chloroplast genome sequence of *F. mandshurica* based on the Illumina pair-end sequencing data (Illumina, San Diego, CA).

A wild individual of *F. mandshurica* was sampled from Shangluo (Shaanxi, China; 108°38′E, 33°26′N) and Voucher herbarium specimens were deposited at the Herbarium of Shangluo University. Genomic DNA was extracted from the fresh leaves using the modified CTAB method (Doyle 1987). Total DNA was used for the shotgun library construction and the subsequent high-throughput sequencing on the Illumina HiSeq 2500 Sequencing System (′). In total, 2.1G raw reads were obtained, quality-trimmed and used for the cp genome assembly using MITObim v1.8 (Hahn et al. 2013) with *F. chiisanensis* (GenBank: NC_037171) (Kim et al. 2018) as the initial reference. The genome was annotated using software Geneious v 9.0.2 (Biomatters Ltd., Auckland, New Zealand). The circular plastid genome map was completed using the online program OGDRAW (Lohse et al. 2013). The annotated chloroplast genome sequence has been submitted to the GenBank under the accession number MF674342.

The complete chloroplast genome of *F. mandshurica* was 155,530 bp in length, which contains a large single copy (LSC) region (86,415 bp), a small single copy (SSC) region (19,279 bp) and a pair of IR regions(25,653 bp). The chloroplast genome of *F. mandshurica* contained 133 genes including 88 protein-coding genes, 37 tRNA genes and eight rRNA genes. In these genes, 16 genes contained one intron and three genes contained two introns. Most of these genes occurred as a single copy; however, 19 gene species in the IR regions are totally duplicated, including eight protein-coding genes, seven tRNA genes and 4 rRNA genes. Out of these 19 gene species, two species (ycf1 and rps12) are partially located within the IR regions, while all the others completely within the IR regions. The overall GC content of *F. mandshurica* chloroplast genome is 37.8%.

In order to determine its phylogenetic status within the family Oleaceae, Phylogenetic analysis of 17 complete chloraplast genome sequences of Oleaceae was performed by MEGA6.0 (Tamura et al. 2013) with *Salvia miltiorrhiza* (Lamiaceae) as the outgroup. As shown in the highly resolved neighbour-joining (NJ) phylogenetic tree (Figure 1), all the species of the family Oleaceae formed a monophyletic clade with a high resolution value and *F. mandshurica* was closely related to *F. chiisanensis* and *F. excelsior.*

**Figure 1.**
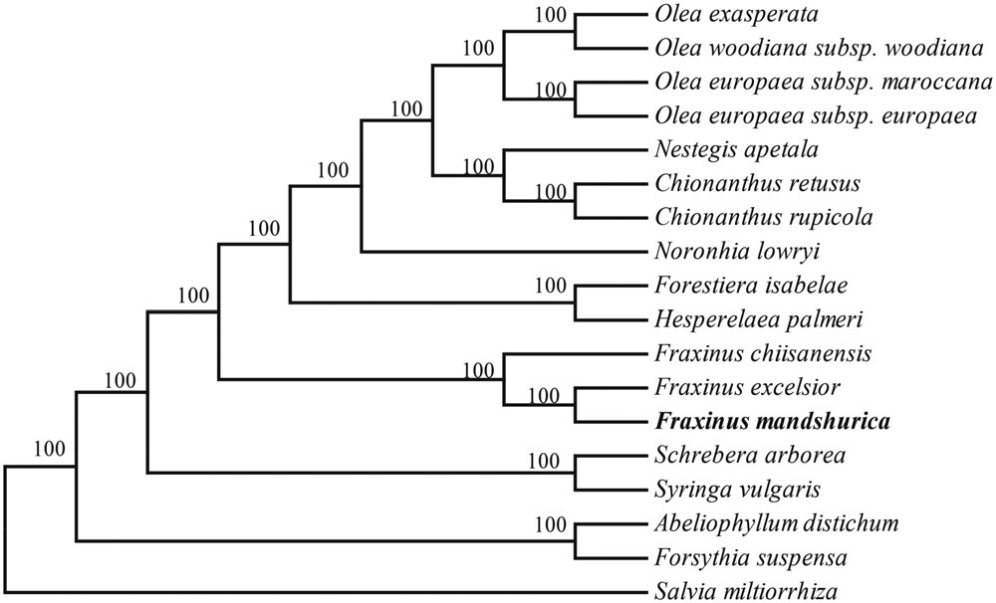
Neighbour-joining (NJ) phylogenetic tree based on 18 complete chloroplast genomes. Accession numbers: *Abeliophyllum distichum* (NC_031445); *Chionanthus retusus* (NC_035000); *Chionanthus rupicola* (NC_036980); *Fraxinus chiisanensis* (NC_037171); *Fraxinus excelsior* (NC_037446); *Forestiera isabelae* (NC_036981); *Forsythia suspensa* (NC_036367); *Hesperelaea palmeri* (NC_025787); *Nestegis apetala* (NC_036983); *Noronhia lowryi* (NC_036984); *Olea exasperata* (NC_036985); *Olea europaea* subsp. *maroccana* (NC_015623); *Olea europaea* subsp. *europaea* (NC_015401); *Olea woodiana* subsp. *Woodiana* (NC_015608); *Schrebera arborea* (NC_036986); *Syringa vulgaris* (NC_036987); *Salvia miltiorrhiza* (NC_020431); *Fraxinus mandshurica* (MF_674342).
